# Editorial: Emerging research: conspiracy beliefs

**DOI:** 10.3389/fpsyg.2023.1325109

**Published:** 2023-11-23

**Authors:** Mark Hallahan, Eric Mayor

**Affiliations:** ^1^Department of Psychology, College of the Holy Cross, Worcester, MA, United States; ^2^Division of Clinical Psychology and Epidemiology, University of Basel, Basel, Switzerland

**Keywords:** conspiracy beliefs, conspiracy theories, conspiracy mindset, narcissism, moral foundations theory, vaccination hesitancy, authoritarianism

People sometimes assert that a powerful group's secret, malign efforts are responsible for bad outcomes even with scant evidence to accept this belief over more plausible explanations. Psychologists' recent interest to understand the consequences of conspiracy beliefs and the antecedent factors that incline people to hold them has made this a rapidly growing research area, especially now as we grapple with the implications of conspiracy theories that are prevalent in public discourse about pressing concerns such as the COVID-19 pandemic (Pilch et al.). One indicator of this area's explosive growth is that Pilch et al. found 274 empirical articles published from 2018 to 2021 meeting the inclusion criteria for their timely and thorough review of recent conspiracy beliefs research. The five articles from this Research Topic represent the key focus of recent conspiracy belief research, which is to understand why people hold conspiracy beliefs and how these beliefs affect their thinking and behavior.

In their review, Pilch et al. find six categories of antecedents of conspiracy beliefs in the literature and a diversity of consequences. The categories of antecedents identified are: cognitive (e.g., analytic vs. intuitive thinking style, relation to other cognitive biases), motivational (epistemic and existential—the two most studied—as well as social motives), personality (including temperament, personality traits, self-evaluations, as well as other dispositional traits), psychopathology (mostly subclinical manifestations of psychiatric disorders; Dark Triad and Dark Tetrad traits), political (ideological orientation, extremist ideology) and sociocultural factors (e.g., Hostede's cultural values, moral foundations, media habits or media consumption, trust in institutions, and religiousness). The authors also examine the consequences of conspiracy beliefs: “The endorsement of conspiracy theories may have a range of negative consequences for both individuals and the society at large” (Pilch et al., p. 9). For the individual, holding conspiracy beliefs can be associated with social stigma and fear of social exclusion, as well as less likelihood to engage in evidence-based prophylaxis (but not pseudo-scientific prevention) and to rely on biomedical treatment. As for social consequences, conspiracy beliefs are also associated with criminal intentions, support for violence, and dehumanization of others. The authors also note conspiracy beliefs might shape political preferences and non-conventional political participation. They note recent increases in the geographical diversity of where conspiracy research is conducted but that it is still heavily reliant on European and North American samples.

Two articles in this Research Topic focus on understanding specific antecedents of conspiracy beliefs. In a sample of adults living in Iran, Nejat et al. find conspiracy beliefs regarding COVID-19 to be associated with religiosity and endorsement of moral foundations of authority and sanctity but not strongly related to Big 5 personality traits. Cosgrove and Murphy examine whether education moderates the association between conspiracy belief and narcissism. Their first study finds that variables related to narcissism (i.e., grandiosity, vulnerable narcissism, need for uniqueness, and need for supremacy) are positively related to conspiracy beliefs. The overall association between conspiracy beliefs and education, including STEM education, are negative, but there also is a surprising moderating relationship such that education predicts higher levels of conspiracy belief for narcissistic individuals. Interestingly, the positive association between need for uniqueness and conspiracy beliefs is only present in highly educated individuals. The authors originally predicted the opposite moderating relationship because education was postulated to be linked to increased critical thinking that would reduce the association of conspiracy beliefs and narcissism. In a second study relying on pre-existing data (*N* = 51,404), the authors examine similar hypotheses in the context of conspiracy belief related to the COVID-19 pandemic: They test the main effects of critical thinking (negatively associated) as well as narcissism and collective narcissism (positively associated) on conspiracy beliefs and further test whether critical thinking moderates the association of conspiracy beliefs with narcissism and collective narcissism. Contrary to the results of the first study and in line with their expectations, the authors find that critical thinking reduces the relationship between collective narcissism and conspiracy beliefs. It could follow that education that fails at increasing critical thinking is not a protective factor in relation to conspiracy belief in narcissistic individuals.

Two other articles in the Research Topic demonstrate the consequences of holding conspiracy beliefs. Romer and Jamieson examine the association between conspiracy mindset and the perceived risk of vaccination of children against COVID-19, as such mindset is grounded in the distrust of governments. In a sample of 1,941 U.S. adults, the authors find strong direct links between conspiracy mindset and endorsement of COVID conspiracies (positive), vaccine misinformation and conspiracies (positive), trust in authorities (negative) and intention to vaccinate against MMR (negative). Perceived COVID risk is negatively associated with trust in authorities, and being vaccinated is negatively associated with vaccine misinformation and conspiracies. Lower risk for child COVID-19 vaccination is predicted by all these intermediary variables, which together explain 76 % of variance in that outcome. Smallpage et al. show that authoritarian tendencies such as social dominance orientation and right-wing authoritarianism are associated with conspiracy beliefs and with other epistemically unwarranted beliefs such as paranormal thinking and belief in pseudoscience. In addition, they posit that conspiracy beliefs may be part of a broader construct that shares common cognitive foundations with these other epistemically unwarranted beliefs.

Conspiracy beliefs are pervasive, consequential, and often difficult to change. Although they have long been part of the human experience, psychologists have only recently turned their attention to understanding these beliefs and why people hold them. The work on this topic has so far been fruitful in identifying factors associated with conspiracy beliefs and the articles in this Special Topic illustrate some of the key lines of inquiry. As a whole, the articles from this special topic synthesize past literature on conspiracy beliefs, provide new scientific evidence of their antecedents as well as examples of their important societal consequences, and suggest directions for further research on conspiracy beliefs.

## Author contributions

MH: Conceptualization, Writing—original draft, Writing—review & editing. EM: Conceptualization, Writing—original draft, Writing—review & editing.

